# Neonatal infections with multidrug-resistant ESBL-producing *E. cloacae and K. pneumoniae* in Neonatal Units of two different Hospitals in Antananarivo, Madagascar

**DOI:** 10.1186/s12879-016-1580-5

**Published:** 2016-06-10

**Authors:** T. Naas, G. Cuzon, A. L. Robinson, Z. Andrianirina, P. Imbert, E. Ratsima, Z. N. Ranosiarisoa, P. Nordmann, J. Raymond

**Affiliations:** Bacteriologie, APHP, EA7361 Univ Paris Sud, Le Kremlin-Bicetre, France; Service de Pédiatrie, Hôpital Tsaralanana, Antananarivo, Madagascar; Service de Néonatologie, Hôpital Soavinandriana, Antananarivo, Madasgascar; Hôpital Begin, Vincennes, France; Institut Pasteur, Antananarivo, Madagascar; Service de Néonatologie, Hôpital Befelatanana, Antananarivo, Madagascar; Medical Microbiology, Fribourg Univ, Fribourg, Switzerland; University Paris Descartes Bacteriologie, Hôpital Cochin, Paris, France

**Keywords:** ESBL, Outbreak, Madagascar, Neonatology

## Abstract

**Background:**

We investigated the molecular mechanism of ß-lactam resistance in extended-spectrum ß-lactamase (ESBL)-producing Enterobacterial strains isolated in neonatal units of different hospitals in Anatnanarivo, Madagascar.

**Methods:**

Bacteria were identified by standard biochemical methods, disc diffusion antibiograms and Etest. Resistance genes were sought by PCR. Strains were characterized by Rep-PCR (Diversilab), plasmid analysis and rep-typing.

**Results:**

From April 2012 to March 2013, 29 ESBL-producing *E. cloacae* and 15 *K. pneumoniae* were isolated from blood culture (*n* = 32) or gastric samples (*n* = 12) performed at day 0 or 2 from 39/303 newborns suspected of early neonatal infection. These infants were treated with expanded spectrum cephalosporins, due to lack of carbapenems, leading to a high mortality rate (45 %). Isolates recovered were all, but 4, multidrug resistant, particularly to fluoroquinolones (FQ) except for 21 *E. cloacae* isolates. Isolates produced TEM-1 and CTX-M-15 ß-lactamases and their genes were located on several self-transferable plasmids of variable sizes sizes that could not be linked to a major plasmid incompatibility group. *E. cloacae* isolates belonged to 6 Rep-types among which two counted for 11 isolates each. The FQ resistant *E. cloacae* isolates belonged to one clone, whereas the FQ susceptible *E. cloacae* isolates belonged to four clones. The *K. pneumoniae* isolates belonged to 9 Rep-types among which one included five isolates.

**Conclusion:**

This study is the first molecular characterization of ESBL-producing isolates from neonatology units in Madagascar, a country with limited epidemiological data. It revealed an important multi-clonal dissemination of CTX-M-15-producing isolates reflecting both the high community carriage and the very early nosocomial contamination of the neonates.

## Background

Extended-spectrum β-lactamase (ESBL)-producing *Enterobacteriaceae* represent a major worldwide threat among drug-resistant bacteria in both hospital and community settings [1]. They are mostly associated with urinary tract infections, but may also cause significant bloodstream-associated infections [1]. ESBLs are often located on large plasmids that also harbor resistant genes to other antimicrobial classes resulting in multidrug-resistant isolates [1, 2]. Plasmid-encoded ESBLs of the CTX-M-type are reported increasingly worldwide in Gram-negative rods and account now for most of the ESBLs found in *Enterobacteriaceae* [1, 3–5]. CTX-Ms form a rapidly growing family that comprises currently up to 154 variants that are divided into five groups according to amino-acid sequence identity (CTX-M-1, −2, −8, −9 and −25 groups) with CTX-M-15 being the most prevalent in most countries [3–5]. *Bla*_CTX-M-15_ genes are often encoded on plasmids belonging to the incompatibility group IncF [2–4]. In the upstream region of CTX-M genes an insertion sequence element, *ISEcp1,* is commonly present and is likely responsible for the transposition process of the genes [6].

Over recent years, the importance of community-acquired infections due to ESBL-producing isolates has been increasingly demonstrated [3–5]. As a consequence, fecal carriage of ESBL-producing isolates is now widely studied in hospitals but also in healthy populations in the community [5]. Surveys since 2000 have shown an alarming trend of associated resistance to other classes of antimicrobial agents among ESBL-producing organisms isolated from community sites [3, 4, 7].

ESBL-producing *Enterobacteriae* were first isolated in Madagascar between 2005 and 2006 from community-acquired urinary tract infections in 9.7 % of isolated *Enterobacteriaceae* [8]. More recently, 21.3 % of clinical isolates from patients in surgery and intensive care units and 21.2 % of intestinal carriage isolates from children hospitalized in a pediatric department of a large teaching hospital were ESBL-producers [9, 10]. The prevalence of carriage of ESBL in the community of Antananarivo was estimated at 10 % in healthy people in 2011 [11]. The most frequenly involved bacteria being *Escherichia coli, Klebsiella pneumoniae* and *Enterobacter cloacae.* More recently, Rasamiravaka et al. reported in 2015, a rate 12 % of ESBL- producing *Enterobacteriacae* in urine samples [12]. The same year, Chereau et al., studying the fecal carriage in pregnant women, reported a rate of 18.5 % of ESBL-producing *Enterobacteriacae* [13].

Infections are a major contributor to newborn deaths in developing countries and are responsible for an estimated 35 % of all neonatal deaths [14]. In resource-poor countries, sepsis due to resistant Gram-negative bacilli is an emerging problem and the currently recommended first-line (penicillin/ampicillin plus gentamicin) or second-line antibiotics (a third-generation cephalosporin) do not provide adequate cover. Laboratory tests are often not available and the diagnosis of neonatal infections is based only on clinical signs leading to an antimicrobial treatment often not adapted to the local epidemiology. For this reason, we decided to conduct a study in Madagascar to identify the epidemiology of bacterial early neonatal infections. This study allowed us to show a predominance of *Enterobacteriaceae* and especially of ESBL-producing *E.cloacae* and *K. pneumoniae*. None of the *Escherichia coli* strains isolated here were ESBL-producers. Therefore, the present work focused on the molecular mechanism of ß-lactam resistance in *E. cloacae* and *K. pneumoniae* isolates identified in these neonatal units of two different hospital at Antananarivo, Madagascar.

## Methods

### Study design

During April 2012 and March 2013, newborns from the two hospitals of Antananarivo with a suspected neonatal infection determined based on a clinical score according to the International and French consensus guidelines (fever (>37°8C) or hypothermia (<35 °C), tachycardia or bradycardia, arterial hypotension, poor perfusion, respiratory distress, apnea, seizure, floppy infant, bulging fontanel, irritability, lethargy, purpura were included [15, 16]. The French guidelines for materno-fetal infections were used to categorize the clinical situations, as proved infection when the blood culture was positive, probable infection when the gastric fluid sample was positive with a pathogen bacteria and a positive C-reactive protein (CRP) [15].

### Bacterial strains, antimicrobial agents and susceptibility testing

Bacterial identification was performed using the API 20E system (bioMérieux, Marcy-l’Etoile, France). Antibiograms for 32 antibiotics (amoxicillin, amoxicillin/clavulanic acid, aztreonam, ceftazidime, cefalotine, cefmandole, cefotaxime, cefepime, cefoxitine, imipenem, meropenem, Moxalactam, pipéracilline, ticarcilline, ticarcilline/acide clavulanique, piperacilline/tazobactam, fosfomycine, colistine, rifampicine, cotrimxazole, ciprofloxacine, pefloxacine; norfloxacine, nalidixic acid, tetracycline, tigecycline, chloramphenicol, kanamycine, amikacine, netilmicine, tobramycine, gentamicine) were determined by the disc diffusion method on Mueller-Hinton agar (BioRad, Marnes-La-Coquette, France) and the susceptibility breakpoints were determined and interpreted as recommended by the Clinical and Laboratory Standards Institute [17]. All plates were incubated at 37 °C for 18 h. Minimum inhibitory concentrations (MICs) of given ß-lactams were determined using the Etest technique (bioMérieux, Marcy l’Etoile, France) for the following *β*-lactam antibiotics: amoxicillin ± clavulanic acid, cefoxitin, cefotaxime, ceftazidime, aztreonam, cefepime and imipenem. Confirmation of ESBL producers was performed by double disc synergy testing between ticarcillin/clavulanate and aztreonam and/or ceftazidime and/or cefepime [18].

### Nucleic acid extractions, PCR and DNA sequencing

Whole-cell DNAs were extracted using QIAamp DNA Mini Kit (Qiagen, Les Ulis, France). The *bla*_CTX-M_, *bla*_SHV_, *bla*_TEM_, minor ESBL and quinolone resistant *qnrA*, *B* and *S* genes were searched for by PCR and subsequently characterized by Sanger sequencing. PCR experiments were performed on an ABI 2700 thermocycler (Applied Biosystems, Les Ulis, France) using laboratory-designed primers [19]. Both strands of the PCR products, were sequenced using laboratory-designed primers with an automated sequencer (ABI PRISM 3100; Applied Biosystems). The nucleotide and the deduced protein sequences were analyzed using software available at the National Center of Biotechnology Information website (http://www.ncbi.nlm.nih.gov).

### Plasmid content, mating out and electroporation experiments

Direct transfer of resistance into azide-resistant *E. coli* J53 was attempted by liquid and solid mating-out assays at 30 and 37 °C [19]. Transconjugant selection was performed on trypticase soy agar plates (bioMérieux) containing ciprofloxacin (3 mg/ml) and either ceftazidime (10 mg/ml) or ticarcillin (150 mg/ml).

Plasmids were introduced by electroporation into *E. coli* TOP10 using a Gene Pulser II (BioRad). Natural plasmids were extracted using Kieser extraction method and subsequently analyzed by electrophoresis on a 0.7 % agarose gel [20].

### Replicon typing

PCR-based replicon typing (PBRT) of the main plasmid incompatibility groups reported in Enterobacteriaceae was performed as described [21] using the respective PBRT controls. The obtained amplicons were sequenced to confirm their identity. Genetic structures surrounding the blaCTX-M gene were determined by PCR using primers specific of the known genetic environment of group 1 CTX-M variants [19].

### Fingerprinting analysis

Genomic relatedness of the *K. pneumoniae* and *E. cloacae* isolates was investigated by semi-automated rep-PCR typing (Diversilab®, bioMérieux) as recommended by the manufacturer. Isolates were considered belonging to a same clone if they shared at least 95 % similarity.

## Results

### Patients

Between April 1st, 2012 and March 31st 2013, 8500 newborn (NB) were born in the two hospitals (Befeletanana and Soavinandriana). Among them, 303 NB (3.6 %) had clinical signs of maternal infections and were included in the study, 164 were from Befelatanana (B) and 139 from Soavinandriana (S). The mean gestational age of the NB was 38 ± 3.5 weeks gestationnal age (WA) comprised between 24 and 43 (Table 1). Out of the 303 NB, 60 (19.5 %) were born before 38 weeks, among which 20 were born before 32 weeks and 40 between 32 and 36 weeks. The sex ratio of the NB was 172 (56.8 %) males and 131 (43.2 %) females. Gastric fluid was sampled from 282 NB, blood culture from 254 NB, and CRP determination from 272 NB. Pregnancy monitoring results are indicated in Table 1.

**Table 1:** ᅟ

	Total *n* (%)	Befelatanana *n* (%)	Soavinandriana *n* (%)	*p*-value
Neonates				
Number	303	164	139	
Sex Male Female				
	172 (56.8)	90 (54.9)	82 (59.0)	
	131 (43.2)	74 (45.1)	57 (41.0)	0.54
Sex-ratio	1.3	1.2	1.4	0.54
Gestational age (weeks) [mean]	38 [24–43]	38 [24–43]	38 [26–42]	0.25
Birth weight (g) [mean, 95%CI]	2663 [2576–2750]	2559 [2441–2677]	2785 [2658–2912]	0.01
Antimicrobial treatment	154 (50.8)	120 (73.2)	34 (24.5)	<0.01
Death	47 (15.5)	41 (25)	6 (4.3)	<0.01
Gastric samples Positive Gastric samples True Positives (contamination excluded) Bacteria *Escherichia coli* ESBL^a^-producer *Enterobacter cloacae* ESBL-producer *Klebsiella pneumoniae* ESBL-producer *Group B Streptococcus* Other organisms^b^				
	282 (93.1)	143 (87.2)	139 (100)	
	60 (21.3)	34 (23.8)	26 (18.7)	<0.01
	72	39	33	
	32 (44.4) 0	15 (38.5) 0	17 (51.5) 0	0.68
	11 (15.3) 7	7 (17.9) 5	4 (12.1) 2	
	6 (8.3) 5	4 (10.2) 3	2 (6.1) 2	
	4 (5.5)	2 (5.1)	2 (6)	
	19 (26.3)	11 (28.2)	8 (24.2)	
Blood cultures Positive blood cultures				
	254 (83.8)	145 (88.4)	109 (78.4)	
True Positives (contamination excluded) Bacteria	76 (29.9)	62 (42.8)	14 (12.8)	<0.01
	99	79	20	
*Escherichia coli* ESBL-producer *Enterobacter cloacae* ESBL-producer *Klebsiella pneumonia* ESBL-producer *Acinetobacter baumannii* *Group B Streptococcus* *Enterococcus sp* Other organisms^c^	7 (7.1) 0	4 (5.14) 0	3 (15) 0	
	28 (28.3) 28	27 (34.2) 27	1 (5) 1	<0.01
	14 (14.1) 11	13 (16.4) 10	1 (5) 1	
	8 (8.1)	6 (7.6)	2 (10)	
	9 (9)	5 (6.3)	4 (20)	
	14 (14.1)	12 (15.1)	2 (10)	
	19 (19.1)	12 (19.2)	7 (35)	
Mothers				
Antibiotic before delivery	36 (11.9)	5 (3)	31 (22.3)	<0.01
Pregnancy monitoring Hospital Health center General practitioner Mid-wife No follow up				
	101 (33.3)	10 (6.1)	91 (65.5)	<0.01
	97 (32)	69 (42.1)	28 (20.1)	
	35 (11.6)	25 (15.2)	10 (7.2)	0.33
	38 (12.5)	33 (20.1)	5 (3.6)	
	22 (7.3)	20 (12.2)	2 (1.4)	<0.01
Intrapartum antibiotic	77 (25.4)	11 (6.7)	66 (47.5)	<0.01

### Bacterial epidemiology

During the study period, 168/254 (66.1 %) blood cultures, 60/282 (21.3 %) gastric fluids, and 102/272 (37.5 %) CRP (cutoff > 6 mg/L) were positive. For 58 NB the CRP was >20 mg/L. Results obtained in each of the two hospitals are summarized in Table 1.

Among the 168 positive blood cultures, 92 were positive with *Staphylococcus coagulase negative* or *Corynebacteriae* and were therefore considered as contaminated. Finally, 76 were positives with 99 bacterial isolates and therefore corresponded to a proven infection. *Enterobacteriaceae* (51/99, 51.5 %) among which *Enterobacter cloacae* 28/51 (54.9 %), *Klebsiella pneumoniae* 14/51 (27.5 %), *Escherichia coli* 7/51 (13.7 %) and *Proteus mirabilis* 2/51 (3.9 %) were the most predominant isolates (Table 1). Group B *Streptococcus, Acinetobacter baumannii* and *Enterococcus sp.* represented respectively 9, 8 and 14 % of the isolates.

Among these 76 positive blood culture, 13 were positive with two bacteria: *Enterobacter cloacae* with *Klebsiella pneumoniae* (five cases), *E. cloacae* with *Enterococcus faecalis* (one case), *Acinetobacter baumanii* was associated with *E. faecalis* or *Streptococcus* sp or *Proteus mirabilis* (three cases), *E. faecalis* was associated with *E. coli* (three cases), and *Staphylococcus aureus* (one case).

Twenty four NB were considered as probably infected since they had a positive gastric fluid culture, a negative blood culture and an elevated CRP according to the French guidelines. Bacteria involved were mostly Gram-negative bacteria (12 *E. coli*, 6 *E. cloacae*, one *K. pneumoniae*, and four group B *Streptococcus* and one *S. aureus*). All other NB were considered as only colonized.

In all, 47/303 (15.5 %) NB died. The mortality rate was 41/164 (25 %) at B and 6/139 (4.3 % at S). Among these, 25 died of neonatal infection (20 with positive blood culture and five with positive gastric fluid and elevated CRP). Among these 25, 24 were born at B of which eight had a positive blood culture with an ESBL-producing *E. cloacae*, four with an ESBL-producing *K. pneumoniae* and two with the both. Others had a positive blood culture with *A. baumannii* (*n* = 1), *Haemophilus influenzae* (*n* = 2), *E. coli* (*n* = 2) and *Streptococcus anginosus* (*n* = 1).

### Bacterial isolates and antibiotic susceptibility

Bacterial isolates and results are summarized in Table 1. Gram negative were the most prevalent bacteria (115/171 isolated bacteria). Among these, 108 were identified as *E. coli* (*n* = 39)*, K. pneumoniae* (*n* = 20)*, E. cloaca*e (*n* = 39), or *Acinetobacter baumannii* (*n* = 10). Looking at the antimicrobial susceptibility, 35/39 *E. cloacae* and 16/20 *K. pneumoniae* were ESBL-producers as revealed by a typical synergy image between clavulanic acid and expanded-spectrum cephalosporins and were selected for further analysis. Their distribution by hospitals is described Table 1. None of the *E. coli* isolates were ESBL-producers.

Antibiograms revealed that all ESBL-producing isolates were multidrug-resistant and most of them were resistant to first line antibiotics (ampicillin, cefotaxime or gentamicin) used to treat neonatal infections. In addition, high rates of resistance to gentamicin (88.9 %) tobramycin (81.5 %), ciprofloxacin (35 %) and to trimethoprim-sulfamethoxazole (87 %), were observed They were susceptible to imipenem while *K. pneumoniae* isolates were also susceptible to cefoxitin. Resistance to cefoxitin in all *E. cloacae* isolates was due to the inducible production of *AmpC* β-lactamase from a chromosomal gene. Among ESBL-producers, 16/17 *K. pneumoniae* and 10/39 *E. cloacae* isolated from blood or gastric samples were resistant to fluoroquinolones (FQR). The finding of multidrug resistance among ESBL-producing isolates is of great clinical relevance due to the limited therapeutic options and the high risk of treatment failure in patients infected with these strains.

### Molecular epidemiology of ESBL-producers

**Fig. 1:**
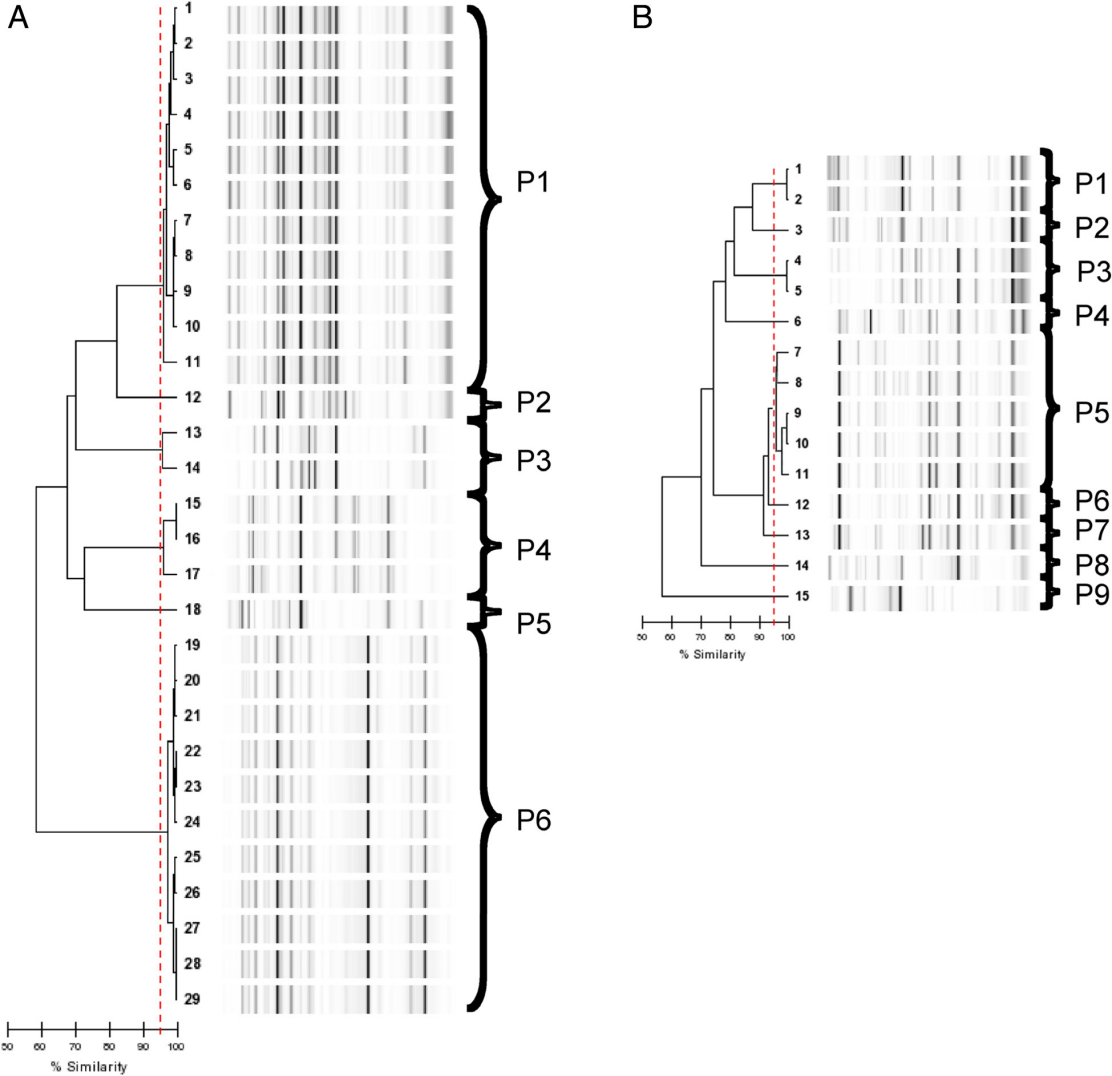
Rep-PCR results showing genetic relatedness between the 29 ESBL-producing *E. cloacae* (**a**) and the 15 ESBL-producing *K. pneumoniae* (**b**) isolates

**Fig. 2:**
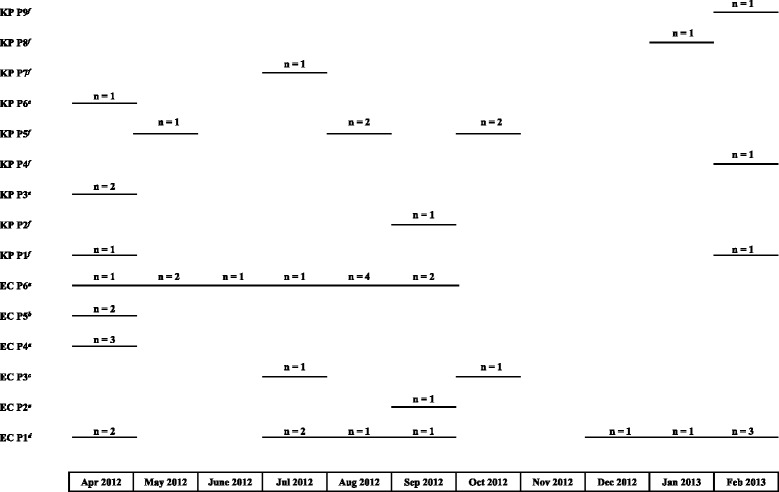
Isolation of the different clones of ESBL-producing *E. cloacae* (*n* = 6) and *K. pneumoniae* (*n* = 9) over the study period. ^*a*^ all strains were isolated at B and were susceptible to fluroquinolones; ^*b*^ all strains were isolated at S, one susceptible and one resistant to fluroquinolones respectively; ^*c*^ all strains were isolated at B, one susceptible and one resistant to fluroquinolones respectively; ^*d*^ all strains were isolated at B, five susceptible and six resistant to fluroquinolones respectively; ^*e*^ all strains were isolated at S and resistant to fluroquinolones; ^*f*^ all strains were isolated at B and resistant to fluroquinolones

Among the 35 ESBL- producing *E. cloacae* 29 were further studied, 21 were isolated from blood culture (20 from B and one from S) and eight were isolated from gastric fluid samples. Eight out of the 29 were resistant to fluoroquinolones. Rep-PCR analyses (Fig. 1) revealed that *E. cloacae* isolates belonged to 6 Rep-PCR-types among which two counted for 11 isolates each. Clone 1 (P1) included 11 strains (one from gastric fluid and ten from blood culture) either resistant to fluoroquinolones (*n* = 7) or susceptible (*n* = 4) and was present all along the study period (Fig. 2). Conversely, clone 6 (P6) included also 11 strains (six from blood culture and five from gastric fluis sample), all resitant to fluoroquinolones and was present for a shorter period of 6 months. One NB exhibited the same strain in blood and gastric fluid and died.

The seven remaining strains were divided into four different clones. Clones 2 (P2), isolated at B was represented each by only one strain from a gastric fluid. Clone 5 (P5) was isolated only at S in gastric fluid and blood from two different NB. In April 2012, three clones were present at the same time at B (Fig. 2).

Among the 16 ESBL-producing *K. pneumonia,* 15 were studied, 11 were isolated from blood culture (ten from B and one from S) and four were isolated from gastric fluid samples. *K. pneumoniae* isolates were of more diverse origin (Fig. 1): they belonged to 9 Rep-PCR-types among 1 (P5) included five isolates sharing more than 95 % similarity. Two other clones (P1 and P3) were represented each by two isolates and the six remaining clones included only one strain (Fig. 1). Clone 1 (P1) included two strains and was present at B hospital at the beginning and at the end of the study. Clone 3 (P3) (including two strains, one from blood culture and one from fluid gastric sample from the same NB) was present only in S hospital. The clone 5 (P5) was present at Befelatanana hospital in May to October. Other isolates were isolated from gastric samples, belonged to three or four different profiles and were all isolated at B.

Interestingly, four blood cultures were positive with both *K. pneumoniae* and *E. cloacae* isolates*.* In two cases, the Rep-PCR profiles were different for both bacteria. In two other cases, the blood cultures isolated from two different NB born two days apart exhibited the same profile for *E. cloacae* (P1) but different profiles for *K. pneumonia*e (P4 and P9).

### Molecular analysis

**Fig. 3:**
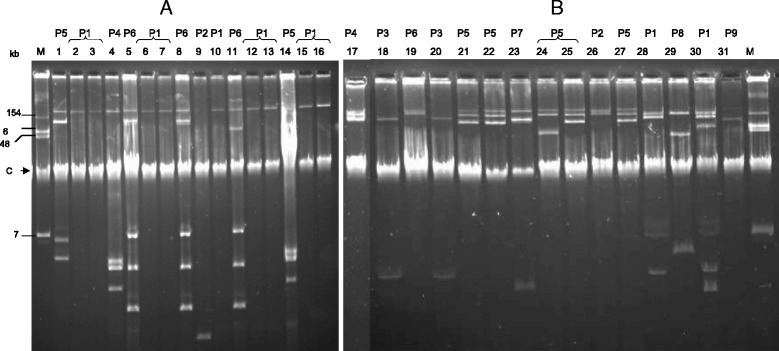
Plasmid analysis by Kieser method and corresponding clones obtained by Rep-PCR (P): (**a**) 15 ESBL-producing *E. cloacae,* 5/6 clones are represented with the two main ones P1 and P6; (**b**) 15 ESBL-producing *K. pneumoniae* isolates, 9/9 clones are represented. M: *E. coli* 50192, reference strain harboring four plasmids (molecular size plasmid marker). “C” indicated the chromosomal DNA band

ESBL producing isolates harboured *bla*_CTX-M_ and *bla*_TEM_ genes as revealed by PCR. Sequencing of the PCR products revealed that *bla*_CTX-M-15_ and *bla*_TEM-1_ genes were present in all isolates. PCR-sequencing of the genetic environment of *bla*_CTX-M_ gene revealed the presence of *ISEcp1* insertion sequence located upstream of all *bla*_CTX-M-15_ gene. The ESBL-producing isolates contained several plasmids of different sizes, ranging from less than 5 kb to more than 150 kb (Fig. 3). Both resistance genes were carried on several self-transferable plasmids of variable sizes. For *E. cloacae* isolates, clone P1 exhibited only one plasmid whereas clone P6 exhibited at least five plasmids of different size. Transformants into *E. coli* DH10B could be obtained with most of the isolates. The *E. coli* transformants had a ß-lactam resistance pattern that correspond to the expression of an ESBL-like gene. Transformants obtained with *E. cloacae* P1 and *K. pneumoniae* P5 were also resistant to gentamicin, tobramycin, tetracycline, cotrimoxazole and chloramphenicol. Transformants obtained with *E. cloacae* P6 harbored the same profile but were susceptible to tetracycline and cotrimoxazole. The *bla*_CTX-M-15_ gene carrying plasmid could also be transferred by conjugation to *E. coli* J53 for all the isolates. PCR-based replicon typing of the main plasmid incompatibility groups reported in Enterobacteriaceae was not able to identify the inc group of the plasmids carrying *bla*_CTX-M-15_ gene. The quinolones resistant isolates were tested negative for qnrA, B and S.

## Discussion

Between April 2012 to March 2013, 35 *E. cloacae* and 16 *K. pneumoniae* isolates producing an ESBL were isolated from blood cultures (*n* = 39) or gastric fluid samples (*n* = 12) performed at day 0 or 1 from 39/165 newborns suspected of early-onset neonatal infection. These NB were treated with ceftriaxone, due to lack of carbapenems, and resulted in a high mortality rate. Molecular studies could be performed on 29 ESBL-producing *E. cloacae* and 15 ESBL-producing *K. pneumoniae.* All strains exhibited *bla*_CTX-M-15_ ESBL gene. The CTXM-15 ESBL is considered to be the most prevalent ESBL worldwide [1, 3, 4]. Our findings confirm the predominance of *bla*_CTX-M-15_ among ESBL producing isolates. Previously in 2009, the *bla*_CTX-M-15_ gene has already been reported to be the most prevalent ESBL in Madagascar, as it was detected in 75.5 % of *Enterobacteriaceae* isolated from feces [11]. These results were confirmed in 2015 showing an increase in the prevalence of colonization by ESBL-producing *Enterobacteriaceae* (18 %, among which 68 % carried the *bla*_CTX-M-15_ gene), consistent with the worldwide increase of ESBL-producing *Enterobacteriaceae* carriage in the community [13]. This predominance is is highlighted by a study involving nine Asian countries reported that *bla*_CTX-M-15_ gene was highly prevalent among ESBL-producing *K. pneumoniae* isolates (60 %, 55/92) [22].

CTX-M genes may disseminate through clonal expansion or horizontal gene transfer [3, 4]. In our study, *ISEcp1* was found upstream from *bla*_CTX-M-15_ at variable distances, as was previously described [7, 23]. *ISEcp1* was found to be in the vicinity of many *bla*_CTX-M_ genes (including *bla*_CTX-M-15_) and was reported to be responsible of the expression of downstream located genes [6]. The plasmids carrying *bla*_CTX-M-15_ were assigned to the IncFII, IncFIA or IncHI2 incompatibility group replicons [2]. Association of the *bla*_CTX-M-15_ gene with IncF plasmids carrying the FII replicon in association with the FIA or FIB replicon has been reported previously for isolates in Canada, France, Spain, Tunisia, and the United Kingdom [2–4, 24]. The IncHI2 plasmid, frequently associated with *bla*_CTX-M-2_ or *bla*_CTX-M-9_, was first identified in *Serratia marcescens* [2], but rarely reported in association with *bla*_CTX-M-15_. In our study *bla*_CTX-M-15_ gene could not be linked to a major plasmid incompatibility group by PCR.

Our study presents some limitations, especially the lack of vaginal samples of the mother prior birth, which would have allowed to distinguish nosocomial and a maternal transmission. The hypothesis of nosocomial infection is more likely with regard *E. cloacae* as two Rep-PCR-types represented each 11 identical isolates from the same hospital, one isolated throughout the study (EC-P1) and another only the first 6 months (EC-P6). The clone EC-P4, including three isolates, was present only in April. Conversely, the *K. pneumoniae* are of much more diverse origin since they belonged to 9 Rep-PCR-types among two included only two isolates and the six other clones only one strain. In two cases, the *E. cloacae* strains isolated both from the gastric sample and blood sample belonged to the same clone (EC-P5 at S hospital and EC-P6 at B hospital).

A community origin and therefore a mother-to-child transmission can then be discussed in this case. This hypothesis was evocated by Cherau et al. who investigated the ESBL-PE rectal colonization among pregnant women at delivery in the community in Madagascar and estimated a prevalence ranging from 14.5 to 22.6 % according to the place [13]. Conversely, the nosocomial hypothesis is supported by the data of a previous study, reporting a clonal outbreak of *K. pneumoniae* harboring *bla*_CTX-M-15_ and *bla*_SHV-2_ genes described in the neonatal units of two hospitals in Antananarivo and highlighting the role of contaminated aspiration tubes [25]. The precocity of neonatal infections due to *E. cloacae* and *K. pneumoniae* occurring between D0 and D1 in this study study does not allow to discriminate between the two hypothesis. It is likely that the two causes of infection have coexisted: the nosocomial hypotheses is more plausible for *E. clocae* (two major clones) and community hypothesis for *K. pneumonia*e. Interestingly, no relationship between the two hospitals has been demonstrated. This is confirmed by the fact that the strains of *E. cloacae* were isolated mainly to the hospital B, except two belonging to a different clone. Interestingly, B hospital supports more deliveries per year (about 7000 vs 1500) with a population of a lower socioeconomic level.

Our study further underscores that in developing countries, neonatal infections are mainly due to Gram-negative bacteria and especially *K. pneumoniae* (respectively 57.4 and 26.4 %) as reported by Zaidi et al. [26]. We found also the same percentage of *A. baumannii* (8 %), Group B *Streptococcus* (8 %) and a lower percentage of *E. coli* (7 %) similarly to previous studies [26, 27].

Neonates are exposed to external risks factors, particularly deficient hygiene that put them at high risk of neonatal infection and if neonatal culture confirmed sepsis rates is of 1–3 per 1000 live births reported from industrialized countries [28], this rate can reached 37 per 1000 live births in developing countries [29, 30]. Poor quality of care in developing countries are a major source of neonatal infections for hospital-born infants. Lack of infection-control procedures, inadequate sterilization of multiuse instruments, understaffing and overcrowded nurseries are responsible for nosocomial infections in most hospitals in developing countries and promotes neonatal infections due to environmental pathogens as reflected in this study by the positivity of the gastric samples cultures with *E. cloacae* and *K. pneumonia*e [31]. A supplementary burden being the high antimicrobial drug resistance rates due to a combination of several factors, including irrational antimicrobial drug use [5]. In addition, imipenem was not marketed in Madagascar at the time of the study explaining, in part, this elevated mortality.

## Conclusion

This study confirmed the global emergence of *bla*_CTX-M-15_ genes from a country with limited epidemiological data. In addition, this is the first molecular characterization of ESBL-producing isolates from Neonatology ward in Madagascar. We confirmed a high prevalence and the multi-clonal dissemination of *bla*_CTX-M-15_ genes among these isolates thus reflecting both high community carriage and a very early nosocomial contamination of the neonates. These findings underline the need for a rational use of antibiotic and for preventive hygiene strategies, which can reduce the burden of neonatal infections in these countries.

## Abbreviations

CRP, c reactive protein; CTX, Cefotaxime; DNA, desoxyribonucleic acid; DO and D1, day zero and day one; ESBL, extended spectrum beta-lactamase; FQ, fluoroquinolones; MIC, minimum inhibitory concentration; NB, newborn; PCR, polymerase chain reaction; Rep-PCR, repetitive extragenic palindromic sequence polymerase chain reaction; WA, weeks gestationam age
